# AMP-Activated Protein Kinase (AMPK) at the Crossroads Between CO_2_ Retention and Skeletal Muscle Dysfunction in Chronic Obstructive Pulmonary Disease (COPD)

**DOI:** 10.3390/ijms21030955

**Published:** 2020-01-31

**Authors:** Joseph Balnis, Tanner C. Korponay, Ariel Jaitovich

**Affiliations:** 1 Division of Pulmonary and Critical Care Medicine, Albany Medical College, Albany, NY 12208, USA; balnisj@amc.edu (J.B.); korpont@amc.edu (T.C.K.); 2 Department of Molecular and Cellular Physiology, Albany Medical College, Albany, NY 12208, USA

**Keywords:** AMPK, protein anabolism, protein catabolism, COPD, muscle atrophy

## Abstract

Skeletal muscle dysfunction is a major comorbidity in chronic obstructive pulmonary disease (COPD) and other pulmonary conditions. Chronic CO_2_ retention, or hypercapnia, also occur in some of these patients. Both muscle dysfunction and hypercapnia associate with higher mortality in these populations. Over the last years, we have established a mechanistic link between hypercapnia and skeletal muscle dysfunction, which is regulated by AMPK and causes depressed anabolism via reduced ribosomal biogenesis and accelerated catabolism via proteasomal degradation. In this review, we discuss the main findings linking AMPK with hypercapnic pulmonary disease both in the lungs and skeletal muscles, and also outline potential avenues for future research in the area based on knowledge gaps and opportunities to expand mechanistic research with translational implications.

## 1. Introduction 

CO_2_ retention in the blood, or hypercapnia, is a frequent comorbidity in patients with chronic pulmonary diseases such as chronic obstructive pulmonary disease (COPD) and others [1,2,3]. It results from a complex combination of decreased ventilatory drive and abnormal lungs’ ventilation–perfusion (V/Q) matching [4,5], and is associated with higher mortality in these patients [1,3,6,7]. Hypercapnia, which typically occurs in advanced forms of COPD [6], causes extracellular acidification [8,9]; that effect is controlled, in the long term, by the renal buffering capacity which keeps the extracellular pH at a relatively normal level. CO_2_ is a highly diffusible species that also regulates, in a pH-independent manner, immune response [10], wound healing [11], edema reabsorption [12], and other processes [2] not influenced by renal compensation. 

Locomotor (non-ventilatory) skeletal muscle dysfunction is also associated with chronic obstructive pulmonary disease (COPD) and other pulmonary diseases [13,14,15,16,17,18]. Muscle wasting leads to worse clinical outcomes including higher disability, re-hospitalization rates and mortality [13,14,16,18,19,20,21]; and results from an imbalance between protein degradation and synthesis, which leads to net muscle loss [21,22]. Over the last few years, our group has established a mechanistic link between elevated CO_2_/hypercapnia and skeletal muscle loss, which is regulated by AMP-activated protein kinase (AMPK)-α2. As CO_2_ retention occurs almost exclusively in the context of pulmonary diseases, this review is focused on the description of AMPK as a sensor of CO_2_ and its implications for pulmonary physiology and physiopathology; and on the mechanisms regulating locomotor muscle turnover driven by CO_2_-AMPK axis. We will also propose future avenues to further investigate the process based on current evidence and knowledge gaps. 

### 1.1. AMPK and Pulmonary Disease

AMPK is a major energy sensor that becomes activated by cellular stress such as low oxygen and glucose deprivation [23]. The mammalian AMPK consists of a heterotrimeric complex formed by a catalytic subunit (α) and two regulatory subunits (β and γ). The phosphorylation of a conserved threonine residue (Thr-172) in the kinase domain of the catalytic subunit is needed for AMPK activation [23,24], a process regulated by upstream kinases, as described in detail in excellent reviews [23,24,25]. Previous research indicates that exposure of alveolar epithelial cells to high CO_2_ induces AMPK phosphorylation, which causes protein kinase C (PKC)-zeta-mediated Na,K-ATPase (sodium pump) endocytosis [12]. This process leads to reduced vectorial sodium and water traffic across the plasma membrane causing pulmonary edema accumulation and further undermining alveolar gas exchange [2,12]. Interestingly, CO_2_ effect on alveolar cells is recapitulated in A549 cells, which are type-II transformed alveolar cells lacking AMPK upstream kinase liver kinase B (LKB1) [12,26], indicating that this kinase is not needed for CO_2_-induced regulation of AMPK in that setting. Moreover, alveolar CO_2_ toxicity is abrogated in alveolar cells previously treated with calcium chelators, or if Ca^2+^/calmodulin-dependent kinase kinase-beta (CaMKK-β) is previously silenced, indicating that CO_2_-AMPK is regulated via calcium signaling in these cells [2,12]. As we discuss later in the text, CO_2_-AMPK interaction is context-dependent as other systems, including skeletal muscle, demonstrate that calcium signaling does not participate in AMPK activation by high CO_2_. Moreover, intracellular reactive oxygen species (ROS), which have been recently shown to be induced by CO_2_ in other contexts [27,28], do not mediate CO_2_-AMPK driven pulmonary toxicity. 

### 1.2. General Concepts about Skeletal Muscle Dysfunction in COPD

**Figure 1:**
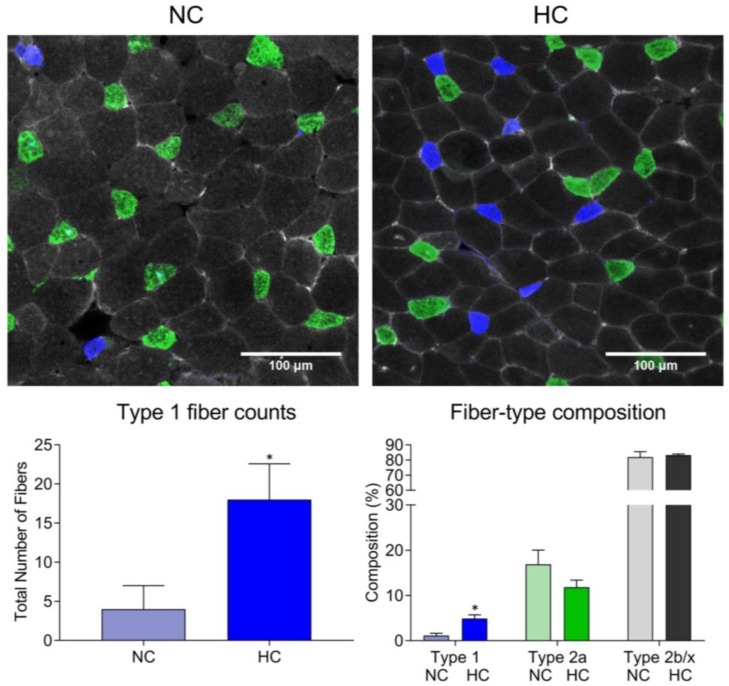
Extensor digitorum longus muscle from 16-weeks old C57 mice housed for 2 months in high CO_2_/normal oxygen (HC) and compared with room air counterparts, as previously described [37,40]. Muscles were frozen and sectioned, and cryosections stained with specific antibodies for isoforms of myosin heavy chain (MyHC): type-1 (blue) and type II-A (green). Unstained fibers (aggregation of type II-B and II-X) were also quantified. As shown by the graph, hypercapnia associates with an increased amount and percentage of type-I fibers. * *p* < 0.05, *n* = 4. Only male mice were used for these experiments.

Muscle dysfunction is a complex process that involves reduction of muscle mass and force-generation capacity [13,21]. In general, muscle mass results from the balance between protein synthesis and degradation which, as presented later in the text, is affected by CO_2_-induced AMPK activation. Maximal force generation capacity is largely influenced by muscle mass [29]. However, submaximal force sustained over time, or endurance, relies on muscle fatigability, which depends mainly on the metabolic properties of individual fibers and not so much on their mass [13,30]. Specifically, oxygen metabolism [31] and calcium handling [32] are main determinants of fiber fatigue-tolerance and critically impact on their submaximal force-generation capacity. Some fibers’ metabolic properties correlate with the expressed isoform of myosin heavy chain (MyHC): Fibers expressing type I are slow-twitched, have predominantly oxidative metabolism and are more fatigue-resistant than type II-expressing fibers [33]. These type-II fibers are referred as fast-twitch and glycolytic. Fiber type I or II classification by MyHC staining is also correlated with calcium sensitivity: Type I fibers are relatively more calcium sensitive than type II fibers and generate a greater fraction of their maximal force for a given amount of mobilized intracellular calcium [32]. There are subtypes of type II fibers—IIa, IIx, and IIb [33]—that are in general less oxidative, calcium sensitive, and fatigue resistant than type I fibers. In COPD locomotor muscles, fiber-type composition typically changes in a process known as “fiber switch” or “transformation,” from type-I to type-II, rendering these muscles metabolically less efficient [34]. The process of fiber switch is contributed by two distinct phenomena: 1) Some fibers undergo selective atrophy, which causes an increase in the relative abundance of the unaffected type [33,34]; and 2) even in the absence of fiber atrophy, the expression of a given MyHC isoform’s gene can be downregulated and a different one upregulated in the same fiber, resulting in a change of its metabolic profile [33]. Fibers’ metabolic properties are not universally correlated with the MyHC isoform expression, as demonstrated by the fact that the expression of oxidative enzymes can be reduced without necessarily a change in the fiber type classification by MyHC isoform expression [35]. Moreover, experiments performed with mice lacking both AMPK β1 and β2 isoforms in skeletal muscle (β1β2M-KO) demonstrate profound reduction of mitochondrial content and exercise-tolerance but no fiber switch [36]. We have shown that chronic CO_2_ exposure leads to a more impactful toxicity on type II fibers in mouse soleus and EDL muscles, although the specific role of AMPK in that process remains to be investigated [37]. AMPK has been shown to participate in the metabolic fate of myoblast under differentiation via fibroblast growth factor 21(FGF21)-induced aerobic phenotype via AMPK. That process operates via SIRT1-AMPK-PGC1α axis [38] and, although it has not been confirmed in the context of CO_2_ stimulation, could represent an attractive mechanism that compensates for the net catabolic effect caused by chronic hypercapnia exposure [39,40]. Interestingly, an increase of type-I (oxidative) fibers in rats exposed to hypercapnia [41] has been reported, and we have recently found that mice chronically exposed to high CO_2_ for 2 months demonstrate higher abundance of type I fibers as well (Figure 1). 

### 1.3. CO_2_-Mediated AMPK Activation Accelerates Protein Muscle Degradation

Insight about the potential CO_2_-induced skeletal muscle toxicity came from observations of Caenorhabditis elegans which demonstrate a skeletal muscle ultrastructural disruption and functional abnormalities in worms kept on hypercapnic conditions [42]. We then exposed adult mice to normoxia-hypercapnia conditions (21% oxygen, 10% CO_2_) which led to a time-dependent reduction of body and muscle weight, and fibers cross-sectional area [40]. As AMPK had been previously implicated in CO_2_ signaling [12] and regulation of muscle turnover [43], to investigate the potential mechanisms linking CO_2_-induced AMPK-activation with muscle loss we exposed differentiated C2C12 cells [44] to normoxia/hypercapnic conditions in a culture medium buffered to maintain normal pH. These cells demonstrated a time-dependent upregulation of phospho-AMPK (Threonine-172), and similar phosphorylation of phospho acetyl-CoA carboxylase (pACC), indicating CO_2_-induced AMPK activation. The same time-course demonstrated reduction of myotubes diameter and induction of muscle ring finger-1 (MuRF1) [40], which is a muscle-specific E3-ligase that regulates proteasomal muscle protein degradation [22,45]. Moreover, MuRF1 knockout (*MuRF1^−/−^*) mice were resistant to the CO_2_-induced muscle loss in comparison with *MuRF1^+/+^* animals. Given that AMPK phosphorylation and MuRF1 induction both associated with reduced myotube diameter, we exposed myotubes previously transfected with siRNA specific for AMPKα1 and AMPKα2 to high CO_2_. AMPKα2 silencing prevented both MuRF1 induction and the reduction of myotubes diameter induced by CO_2_ exposure. In response to metabolic stress, AMPK has been shown to control transcriptional activity via FoxO3 [46]. Thus, we investigated that transcription factor as a potential link between elevated CO_2_ and muscle loss, and demonstrated that silencing of FoxO3 prevents the hypercapnia-induced MuRF1 expression and reduction of myotubes diameter; and specifically that overexpression of FoxO3 constructs holding serine-to-alanine mutations in the six residues known to be targeted by AMPK [46] also abrogates the muscle catabolic process. In that research, we exposed mice to 3 weeks of high CO_2_ and did not appreciate a fiber-type specific effect. As presented below, longer exposure to hypercapnia causes a reduction of fibers’ cross-sectional area that is more pronounced in type-II (glycolytic) fibers [37]. 

### 1.4. CO_2_-Mediataed AMPK Activation Attenuates Muscle Protein Synthesis

Previous evidence from our laboratory suggested that C2C12 myotubes exposed to elevated CO_2_ and normal oxygen demonstrated a reduced anabolism [40]. Further experiments demonstrated that the incorporation of the amino acid puromycin to the myotubes—a surrogate of protein synthesis [47]—was severely reduced in CO_2_-exposed cells [37]. Deaccelerated protein synthesis can be due to either decreased synthesis rate, reduced ribosomal biogenesis, or a combination of both. Ribosomal biogenesis involves the generation and processing of the four ribosomal RNA (rRNAs) and more than 80 ribosomal proteins that form the mature 80S eukaryotic ribosome [48]. Three classes of RNA polymerases participate in that process, which also requires the synthesis of an array of proteins related to processing, assembly, and nuclear import/export of ribosomes [49]. Synthesis of rRNA is a major rate-limiting step in ribosomal biogenesis, with rRNA comprising 85% of total cellular RNA [50]. Specifically, three of the four rRNAs (28S, 18S, and 5.8S rRNAs) are transcribed from a single gene (ribosomal DNA; rDNA) that exists in hundreds of tandem repeats throughout the genome [51]. Transcription of rDNA via RNA polymerase 1 (Pol1) leads to the generation of a precursor rRNA, 45S pre-rRNA, which is processed to form the 28S, 18S, and 5.8S rRNAs. 

A large-scale analysis of muscle proteome from hypercapnic animals indicated that high CO_2_ is associated with reduction of critical elements of protein translation, and with an ontology term describing reduced structural constituents of the ribosome [37]. Moreover, our data demonstrate hypercapnia leads to depressed ribosomal biogenesis in human and mice muscles, and reduced protein synthesis in-vivo and in two independent skeletal muscle cell lines in-vitro [37]. These processes are regulated by AMPKα2 (but not AMPKα1) as demonstrated by the prevention of CO_2_-induced depressed ribosomal biogenesis and puromycin incorporation in both primary and C2C12 myotubes [37]. Although transcription factor TIF1-A has been shown to mediate the effect of AMPK on ribosomal gene expression [37,52], silencing of that gene was unable to prevent the effects of high CO_2_ on protein synthesis. We confirmed the specificity of that silencing by tagging TIF1-A gene with Crispr-Cas9-mediated 3X-flagging and siRNA technology. Similar lack of effects was demonstrated with previous silencing of lysin demethylase KDM2A, which has also been shown to regulate ribosomal gene expression via AMPK in cancer cells [53]. 

Independently of ribosomal biogenesis, AMPK-regulated protein synthesis rate is also controlled via mTOR pathway. However, that process seems to be not relevant in the context of hypercapnia given that while myotubes treated with rapamycin demonstrated a robust dephosphorylation of mTOR, no difference in mTOR phosphorylation was observed in the context of CO_2_ stimulation [37]. Moreover, while high CO_2_ causes robust and significant downregulation of 45s-Pre-RNA expression and puromycin incorporation, rapamycin exerts no significant effect in 45s-pre-RNA yet it causes a significant reduction of puromycin incorporation [37]. Thus, CO_2_ leads to attenuated protein synthesis via AMPKα2 via depressed ribosomal gene expression and independently of the canonical mTOR pathway. 

### 1.5. Other Effects of CO_2_ on Skeletal Muscle Potentially Regulated by AMPK

Although muscle turnover in COPD has been mainly attributed to the balance of intracellular protein synthesis and degradation [13,21,54], recent interest has emerged in the process of myogenesis, which is muscle turnover contributed by myogenic stem (satellite) cells [55]. These cells contribute to muscle homeostasis in the context of organ development and injury-repair cycles [56]. Injurious events crucially occur in COPD in the setting of exacerbations and infections [57,58], which lead to acute decompensations for limited periods of time after which patients typically fail to recover the baseline status they had before the acute event [59]. Indeed, frequency and severity of COPD exacerbations and infections powerfully associate with loss of muscle and lung integrity, and with higher mortality over time [59,60,61]. In stable conditions, adult muscle satellite cells remain in a quiescent state, which means that they are maintained in a G0 phase that can be re-engaged in cell proliferation upon activation (that potential capacity differentiates quiescent from terminally differentiated and from senescent G0 states). Satellite cells activation leads to two distinct consecutive stages: A symmetrical cell division phase that expands the satellite cells pool in order to produce myogenic cells and also repopulate the pool of cells needed to return to quiescence; and asymmetrical division, which commits cells to the myogenic differentiation [56]. There is not research done in the field myogenesis in the context of CO_2_ retention. However, we have observed that chronically hypercapnic mice demonstrate evidence of nuclear centralization [40], a histological hallmark of muscle repair after injurious events which typically require satellite cells involvement. Moreover, our previous analysis of muscle proteome from animals exposed to chronic hypercapnia reveals “ATP binding” (GO:0005524) as the most downregulated ontology term [37]. As this term is highly enriched in bioenergetics-related genes, that proteome suggests possible bioenergetic effects induced by high CO_2_. Because CO_2_ is a highly diffusible species (~20 times higher diffusibility across biological membranes than O_2_) [9,62], it is likely that hypercapnia’s effects appreciated on the muscle fiber are also impactful on the satellite cells’ microenvironment. Preliminary data from our lab demonstrates a CO_2_-induced C2C12 cells reduction of 5-ethynyl-2′-deoxyuridine (EdU) incorporation, which is a standard readout of symmetrical satellite cells division [63]. Importantly, hypercapnia has been shown in the past to cause a reduction of cell proliferation due to mitochondrial dysfunction [11], and AMPK has similarly been shown to regulate cell cycle arrest via phosphorylation of p53 [64]. Moreover, AMPK has been shown to be critical for myogenesis by causing a Warburg-like glycolysis during the repose to muscle injury which is indispensable for muscle regeneration [65]. Moreover, the upstream kinase LKB1 has also been shown to be indispensable for myogenesis both during development and regeneration [66]. These data support the rationale for investigating the potential link between CO_2_ exposure, AMPK activation, symmetrical cell division, and myogenesis. One intriguing aspect that remains to be elucidated is the fact that AMPKα1 is the isoform expressed in undifferentiated satellite cells [65], and skeletal muscle turnover modulated by CO_2_ has so far been shown to be specifically mediated by AMPKα2. Thus, it will be interesting to define whether myoblast AMPKα1 isoform is responsive to elevated CO_2_ or if the effects of hypercapnia in these cells operates via an alternative pathway. It is also possible that CO_2_ toxicity during myogenesis occurs via AMPKα2 at later stages of differentiation—after satellite cells commitment—when that isoform becomes expressed. 

## 2. How is AMPK Regulated by CO_2_? 

**Figure 2:**
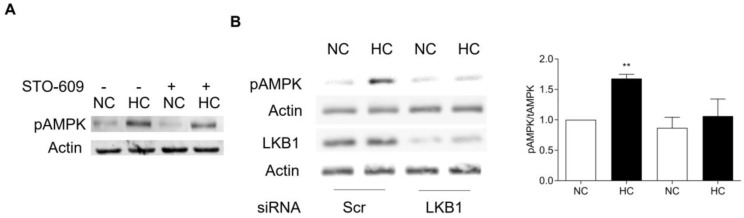
(**A**): C2C12 cells were differentiated with 2% horse serum-supplemented media to form myotubes and then exposed to normo and high CO_2_ in the presence of calmodulin kinase inhibitor STO-609 for 24 h. Then, cells were lysed, and equal amount of total protein was used for SDS-PAGE and immunoblotted using phospho-AMPK antibody. Actin (Sigma, #5316) was used as a lane-loading control as previously reported [37,40]. There was no effect of STO-609 on the CO_2_-induced pAMPK upregulation; *n* = 3. (**B**): Similar C2C12 myotubes were transfected with scramble or specific LKB1 siRNA (Santa Cruz Biotechnology, #35817). Twenty-four hours later, cells were exposed to normo and high CO_2_ and then, were processed for immunoblotting using phospho-AMPK antibody. Actin was used as a lane-loading control as previously reported, and LKB1 (Cell Signaling, #3047) and actin (on the same blot) antibodies were used as transfection control. LKB1 silencing prevented the CO_2_-induced pAMPK upregulation. ** *p* < 0.01, *n* = 3.

It remains unclear how hypercapnia activates cell signaling in different cellular settings including drosophila [67,68], *C. elegans* [42], and mammals [2,11,12,37,40]. Different tissues on the same organism operate through overlapping but distinct pathways. For example, pulmonary alveolar fluid reabsorption is regulated via a CO_2_-AMPKα1 mechanism [2,12], whereas muscle protein turnover is controlled by CO_2_-AMPKα2 [37,40]. Hypercapnia-induced fibroblasts-reduced proliferation operates independently of AMPK and via microRNA-183 (miR-183)-regulated isocitrate dehydrogenase 2 (IDH2) [11]. In the specific case of skeletal muscle, we found that Ca^2+^/calmodulin-dependent protein kinase kinase (CaM-KK) inhibitor STO-609 is unable to abrogate CO_2_-induced AMPK phosphorylation, whereas previous silencing of LKB1 fully prevents that activation (Figure 2). These data are consistent with the fact that AMPKα2 is the main isoform expressed in skeletal muscle [69], which is relevant for maintenance of muscle function [70] and mass [71]; and has also been shown to be relatively more sensitive to AMP/ATP ratio in comparison with AMPKα1 [72]. 

It remains undefined whether the effects of hypercapnia on skeletal muscle are universal or depend on the underlying disease leading to CO_2_ retention. For example, it is unclear whether patients with CO_2_ retention in the context of obesity hypoventilation syndrome (OHS) develop significant muscle atrophy or not. OHS patients demonstrate decreased levels of AMPK activator adiponectin [73], which could lead to reduction of the CO_2_-AMPK pathway and thus antagonize hypercapnia-induced muscle catabolism. Activity of AMPK increases in the post-bariatric surgery period [24], indeed indicating that obesity is associated with lower AMPK signaling. Additionally, as hypercapnic COPD leads to a stable CO_2_ retention, while OHS is associated with intermittent episodes, these two entities could be associated with qualitatively different high CO_2_ exposures [74,75] and muscle turnover response. For example, change from endurance to resistance exercise was reported to cause divergent effects on muscle AMPK activation and protein synthesis, despite similar total exercise intensity [76]. Thus, CO_2_ stimulation quality, even at similar quantitative total exposure, could cause dissimilar muscle effects on AMPK and contribute to different muscle phenotypes in COPD versus OHS. The lack of a well-established animal model of hypercapnia COPD (see below) has so far precluded define the CO_2_-induced signaling in a disease specific context. Moreover, the different effects of exercise on AMPK activation could have important clinical implications for the implementation of rehabilitation protocols in CO_2_-retaining COPD patients [29,77] given the harmful effect of CO_2_ on muscle AMPK-regulated turnover. Recent studies have indicated that COPD patients undergoing different types of rehabilitation exercises activate distinct pathways [78], which likely impacts on the protein turnover potential entailed by these strategies. 

### 2.1. Interactions Between CO_2_ Retention, AMPK, and Autophagy

AMPK is one of the most important autophagy regulators [79]. Autophagy is dysregulated in locomotor muscles from COPD patients [80], yet its relevance is unclear [81]. While seminal research established that autophagy integrity supports muscle mass [82], recent data suggest that inhibition of autophagy prevents COPD muscle fibers’ atrophy [83]. Moreover, while some authors reported that muscle mRNA levels of BECLIN1 and LC3B are not significantly different among COPD versus healthy controls [84], others have shown increased LC3B-II (marker of autophagosomes formation), BECLIN1 and SQSTM1 (p62) protein levels in muscle biopsies of COPD patients [80], suggesting that autophagy is significantly induced in this setting. Therefore, it remains undefined if dysregulated autophagy in COPD skeletal muscle represents global downregulation, upregulation, or deacceleration of autophagy flux with preservation of other regulatory components [85]. It is also unclear whether one or more autophagy specific steps such as nucleation [86] or lipidation [87] are affected, or whether there are different phenotypes variably expressed in the COPD population [78,88]. COPD-driven autophagy dysregulation could be mediated by oxidative stress signals, which are ubiquitous in that population [89] and known to undermine critical autophagy steps including inhibition of Atg7 [90]. While we have shown that CO_2_ retention causes AMPK activation, mTOR dephosphorylation at serine 2448, which is a master pathway switch [91], is not regulated by CO_2_ either in cultured C2C12 cells or in mouse skeletal muscle in vivo [37]. Therefore, while it would be possible that CO_2_-induced AMPK activation modulates autophagy via an alternative route including Serine 317 and Ser 777 phosphorylation of Ulk1 [92], it is also conceivable that a context-specific process limits the effects of CO_2_ on autophagy via AMPK, which could be defined if an animal model of CO_2_-retaining COPD-induced muscle wasting became available (see below). 

### 2.2. Limitations and Future Avenues to Investigate the Process of CO_2_-Induced Muscle Dysfunction

AMPK signaling has been found upregulated in locomotor muscle from patients with COPD [80,81], although clinical studies focused on hypercapnic patients are scarce due to the difficulties obtaining muscle biopsies from patients with CO_2_ retention—these patients tend to be the sickest in the COPD population. Hypercapnia does not occur in a vacuum but in the context of a specific disease process that has unique characteristics and covariables impacting biological processes. While we have recently reported mechanisms regulating muscle atrophy in hypercapnia [37,40], and previous research demonstrates relevant effects of other signals such as hypoxia and immobilization [93,94]; until now there was no animal model of COPD-driven muscle dysfunction that aggregates different aspects present in humans [95]. Information available on this process has been largely observational or supported by models consisting of single stimulus effects on otherwise healthy mice [77,96,97]. These reductionist models contribute powerful data, yet the addition of a comprehensive “syndromic” model of COPD in which muscle dysfunction occurs as a comorbidity would be very valuable to explore individual signals’ effects in clinically meaningful contexts. Mechanistic research focused on COPD-induced muscle dysfunction, and the effect of CO_2_ retention in that context, requires developing a sophisticated animal model that ideally should fulfill the following conditions: 1) Be inducible, in order to minimize temporal confounders such as muscle development and age-related sarcopenia [98,99]; 2) be robust enough and slowly-developing, to reminisce a level of disease severity and chronicity shown by the majority of COPD patients with muscle dysfunction [100]; 3) develop the muscle phenotype after, and not simultaneously with, the occurrence of pulmonary disease [101,102]; 4) recapitulate features observed in humans including morphologic, metabolic, and functional aspects of muscle dysfunction [103]; and 5) develop muscle dysfunction in the context of COPD and not due to a single stimulus to an otherwise healthy mouse. That animal model would represent a bottom-line to understand complex interactions occurring in patients suffering from COPD-associated muscle dysfunction. Analysis of specific mechanisms driven by single stimuli can be performed independently, and eventually merged with a syndromic model such as the one we recently reported, contributing clinically to more meaningful data. We have investigated skeletal muscle dysfunction in a previously established mouse model of pulmonary emphysema [104], which fulfills all the mentioned criteria: Condition #1: doxycycline-inducible, Club cell-targeted interleukin 13 overexpression (*IL13^TG^*); condition #2: a very robust pulmonary phenotype with significant hypoxemia (mean saturation ~77% at room air) but not chronic CO_2_ retention (HCO_3_^−^ 23.6± 2.3 mEq/l); condition #3: a consistent trajectory of weight loss that occurs after emphysema development; condition #4: reduction of muscle mass, force-generation capacity; and metabolic dysfunction as shown by COPD patients [105]. This model deliberately does not involve cigarette smoking as this exposure causes muscle toxicity independently of pulmonary disease [106,107,108] (contradicts condition #3), leads to minimal weight and muscle loss [97] (contradicts condition #4), and represent a single stimulus to an otherwise healthy animal (contradicts condition #5). Future research in which this or other validated models of COPD-induced muscle wasting are exposed to chronic hypercapnia could produce data with more translational value and define the role of AMPK in this process. Moreover, analysis of diverse signaling pathways triggered by chronic hypercapnia in different clinical settings such as COPD and OHS could allow elucidation of common signatures and diverse, context dependent processes. These analyses could address the intriguing and unresolved question of whether CO_2_ retention causes one or multiple “*hypercapnias*”. 

## 3. Conclusions

**Figure 3:**
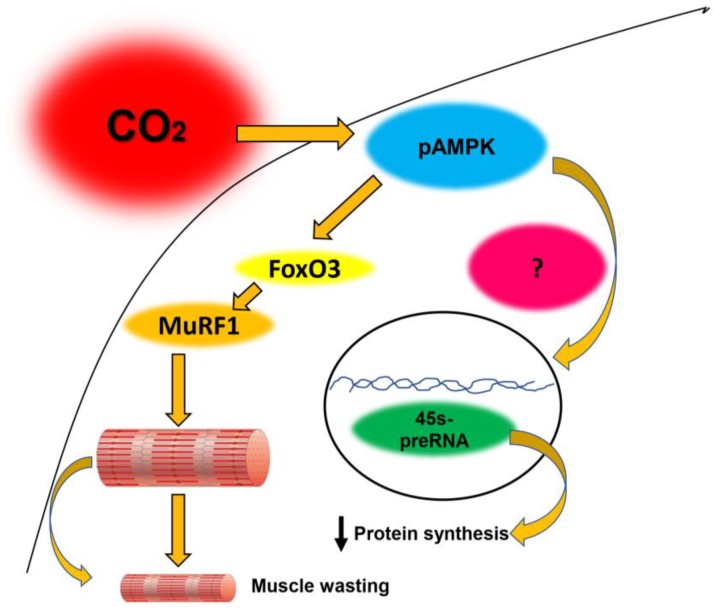
Schematic representation of the dual effect of CO_2_ retention on protein anabolism (suppression) and catabolism (acceleration). See details in references [37,40].

CO_2_ retention or hypercapnia activates multiple signaling pathways in diverse species and tissues. In skeletal muscle, we have found that hypercapnia regulates muscle anabolism and catabolism via AMPKα2, which is of pathophysiological relevance for patients with chronic CO_2_-retaining pulmonary diseases. The lack of validated animal models of pulmonary disease induced muscle dysfunction has so far precluded analyzing the muscle-specific effects of CO_2_ in the context of a particular disease. Our recently established animal model of COPD-induced muscle dysfunction will be instrumental in defining whether CO_2_ retention leads to complex interactions that potentiate muscle wasting, with and without AMPK regulation. A summary of the mechanisms regulating CO_2_-induced skeletal muscle dysfunction is presented in Figure 3.
